# Transactions of the Homœopathic Medical Society of the State of New York

**Published:** 1889-03

**Authors:** 


TRANSACTIONS OF THE HOMŒOPATHIC MEDICAL SOCIETY OF THE STATE OF NEW YORK. Volume XXIII. 1888.

A neatly printed volume of 370 pages, with much of the same routine reports which characterize the "work" of most medical societies. The President, Dr. J. H. Paine, indulges in a tiresome harangue upon the iniquities of dynamization and of the deluded physicians who use such vagaries. The Bureau of Materia Medica, under Dr. Van Denburg, gives a study of Belladonna.

